# Supplementary figures and images for: Circulating MicroRNAs Highly Correlate to Expression of Cartilage Genes Potentially Reflecting OA Susceptibility—Towards Identification of Applicable Early OA Biomarkers
Source: Biomolecules. 2021 Sep 13;11(9):1356. doi: 10.3390/biom11091356 (PMC8468331; doi:10.3390/biom11091356)

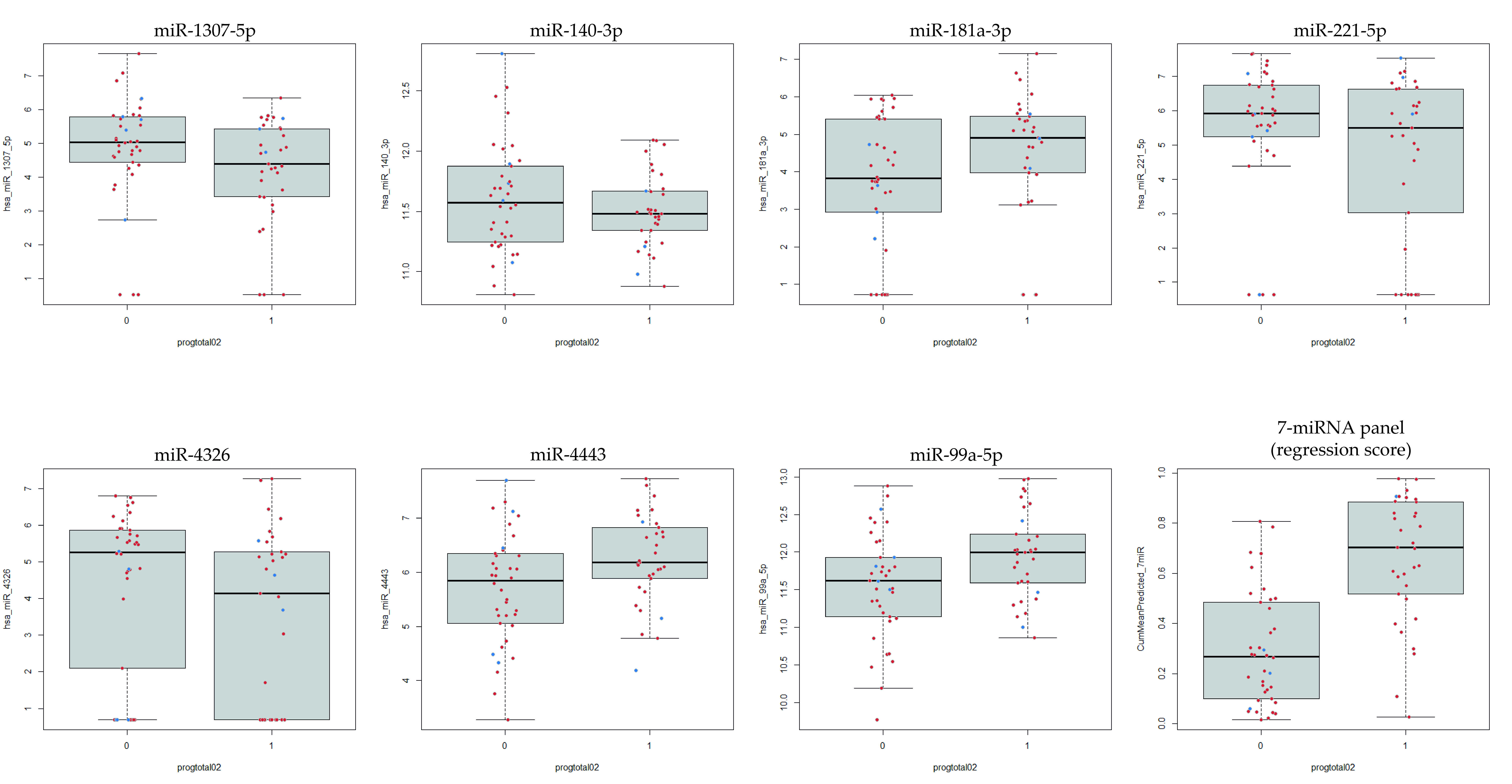

Supplement: Supplementary file 1 [file biomolecules-11-01356-s001.zip › biomolecules-1325009 supp for proof.tif]
